# Compensatory Hippocampal Neurogenesis in the Absence of Cognitive Impairment Following Experimental Hippocampectomy in Adult Rats

**DOI:** 10.3389/fncel.2021.709291

**Published:** 2021-08-31

**Authors:** Giuliana T. M. Cardoso, Walace Gomes-Leal, Edna C. S. Franco, Antonio Pereira, Francinaldo L. Gomes, Ana Leda F. Brino, Silene M. A. Lima

**Affiliations:** ^1^Laboratory of Neurobiology, Institute of Biological Sciences, Federal University of Pará, Belém, Brazil; ^2^Laboratory of Experimental Neuroprotection and Neuroregeneration, Institute of Biological Sciences, Federal University of Pará, Belém, Brazil; ^3^Pathology Unit, Evandro Chagas Institute, Ananindeua, Brazil; ^4^Laboratory of Signal Processing, Institute of Technology, Federal University of Pará, Belém, Brazil; ^5^Neurology Unit, João de Barros Barreto University Hospital, Federal University of Pará, Belém, Brazil; ^6^Experimental School of Primates, Center for Theory and Behavioral Research, Federal University of Pará, Belém, Brazil

**Keywords:** epilepsy, adult neurogenesis, neuroplasticity, hippocampectomy, memory

## Abstract

Temporal lobe epilepsy (TLE) is the commonest type of focal epilepsy in adult humans, and hippocampal sclerosis (HS) is the main pathological finding in this type of epilepsy. In refractory TLE, patients are indicated for unilateral resection of the affected hippocampus by a surgical procedure called hippocampectomy which generally does not cause any cognitive impairment. Once adult hippocampus is a region of endogenous neurogenesis, even in elderly people, we have hypothesized that a compensatory increase in hippocampal neurogenesis might occur in the remaining hippocampus after unilateral hippocampectomy. To test this hypothesis, we performed unilateral hippocampectomy in adult Wistar rats, which were perfused at 15 (G15) and 30 (G30) days post-surgery. Eighteen Wistar rats were randomly distributed in the following experimental groups: control (no surgery, *N* = 6), G15 (*N* = 6), and G30 (*N* = 6). Adjacent cortex and hippocampus of the left hemisphere were completely removed. Behavioral procedures were performed to address possible cognitive impairments. Brains were collected and fixed from animals belonging to all experimental groups. Gross histopathology was performed using thionine staining. Neuroblasts and mature neurons were immunolabeled using anti-doublecortin (DCX) and anti-NeuN antibodies, respectively. Numbers of DCX and NeuN positive cells were quantified for all experimental groups. Animals submitted to hippocampectomy did not present any cognitive impairment as evaluated by eight-arm radial maze behavioral test. The remaining hippocampus presented a higher number of DCX positive cells compared to control (*p* < 0.001, ANOVA-Tukey) at both G15 and G30. A higher number of NeuN positive cells were present in the granular layer of dentate gyrus at G30 compared to control and G15 (*p* < 0.001, ANOVA-Tukey). The data suggest that unilateral hippocampectomy induces compensatory neurogenic effect in the contralateral hippocampus. This may underlie the reported absence of significant cognitive impairment and parallels the findings in human patients submitted to unilateral hippocampectomy to treat refractory TLE.

## Introduction

Epilepsy is a chronic neurological disorder affecting nearly 1–2% of the world population, and its hallmark is an abnormal increase in the predisposition to seizures (Fisher et al., 2005). Among epilepsies, temporal lobe epilepsy (TLE) is the most common type of refractory epilepsy in adult humans; in TLE, the epileptic focus is located in the temporal lobe, and, in about 50% of cases, in the hippocampus (Anyanwu and Motamedi, 2018). Unilateral amygdalohippocampectomy is often effective in seizure control.

It has been widely reported that bilateral damage to the hippocampal formation may produce impairments in memory consolidation in humans (Squire, 2009; Squire and Wixted, 2011). However, patients with TLE submitted to unilateral hippocampectomy usually do not present cognitive deficits (Vojtěch et al., 2012; Jobst and Cascino, 2015; Anyanwu and Motamedi, 2018).

There is a continuous production of new neurons in the adult hippocampus both in experimental animals (Kempermann et al., 2015; Lima and Gomes-Leal, 2019) and in the human brain, and in older people (Boldrini et al., 2018; Lima and Gomes-Leal, 2019; Moreno-Jiménez et al., 2019). The existence of a compensatory neurogenic mechanism might underlie the fact that TLE patients do not present observable cognitive deficits related to memory consolidation.

In this study, we performed experimental unilateral hippocampectomy in adult rats to investigate possible cognitive impairments and whether compensatory neurogenic events occur in the remaining hippocampus.

## Materials and Methods

### Animals

Eight-weeks-old male adult Wistar rats (*Rattus norvegicus*, *n* = 18) breaded and donated by the Federal University of Pará (UFPA) central animal house were used in this study. Procedures were evaluated and approved by the Ethics Committee on Research with Experimental Animals of the UFPA (CEUA-UFPA) (approval number 6552280616). Procedures for the use of experimental animals suggested by the Society for Neuroscience, by the US National Research Council’s Guide for the Care and Use of Laboratory Animals, by the US Public Health Service’s Policy on Humane Care and Use of Laboratory Animals, by the Guide for the Care and Use of Laboratory Animals, and by CEUA-UFPA were followed.

Animals were maintained in the animal facility at the Laboratory of Experimental Neuroprotection and Neuroregeneration in individual cages with a controlled temperature at 23°C, a light/dark cycle of 12 h, and water and food *ad libitum*.

To test the hypothesis that has been raised, three experimental groups were delineated: animals submitted to surgical removal of the left hippocampus (unilateral hippocampectomy) with survival times of 15 (G15) and 30 (G30) days. This period was chosen based on the time of neuroblast formation and neuronal maturation in both striatum (Arvidsson et al., 2002) and hippocampal dentate gyrus (van Praag et al., 2002) after acute brain injury in rodents. Animals in the control group were not submitted to any surgical procedures (sham animals). We used the normal rate of test learning by sham animals as parameter applied to behavioral test performance.

To evaluate whether the sample size of animals used in this study is suitable for statistical inferences, we applied the resource equation method (Festing, 2006). According to this method, E = total number of animals – total number of groups. A number between 10 and 20 means no additional animals are required according to the 3R principles. A number higher than 20 means that an additional number of animals are required to increase the statistical power. Applying this equation to the sample, we have obtained E = 18 – 3 = 15. As number 15 is between 10 and 20, we infer, according to this analysis, that number 6 per group is suitable for the present analysis following the 3R principles.

### Surgical Procedures

Animals were anesthetized with intraperitoneal injections of xylazine hydrochloride (10 mg/kg) and ketamine hydrochloride (80 mg/kg). Surgery was performed only after complete abolishment of both corneal and paw withdraw reflexes. If necessary, additional anesthetic doses were administered with dose adjustment one-third of the initial dose.

The animal was placed in the stereotaxic apparatus and a longitudinal surgical incision was made for the exposure of the skull and visualization of bregma and sagittal sutures. Coordinates for the location of the dorsal hippocampus were adjusted according to the stereotaxic atlas by Paxinos and Watson (1998). We used the following stereotaxic coordinates in relation to the bregma: −1.5 mm in the midlateral direction (M/L) and + 2.8 mm in the anterior-posterior direction (A/P). With the use of a dental drill, craniotomy was performed in the region around the hippocampus reference area (indicated by the stereotaxic coordinates). Under stereo surgical microscope observation, meninges and adjacent cortex were removed for exposure of the dorsal hippocampus, which was individualized and removed in blocks. A hemostatic powder was used to minimize bleeding. At the end of the procedure, skin and muscles were sutured and an anti-inflammatory ointment was administered to assist the healing process. Antibiotic was administered to the animal for 3 days at 24-h intervals.

### Behavioral Procedures

An eight-arm radial maze was used to evaluate whether the surgical intervention produces learning and memory deficits in the experimental animals. The eight-arm radial maze consists of an octagonal platform with a diameter of 34 cm, from which eight arms, 47.5 cm long and 13 cm wide, are placed at a height of 60 cm from the ground. Before the start of the test, the animals were submitted to a food restriction period (around 4 days) in which they had a decrease in the amount of feed. At 24 h before the test onset, animals were submitted to food deprivation.

The behavioral procedures were initiated in the experimental groups G15 and G30 at 8 and 23 days post hippocampectomy. Behavioral testes were finished at 15 and 30 days post-surgery. Animals in the control group were not submitted to surgical procedures.

Animals were exposed for 7 days to the maze. During this period, rats explored the maze (once a day per animal). Every day, four specific arms of the maze were baited with a piece of rat food, always in the same four arms. For each exploration day, animal was placed on the central platform and allowed to freely explore the apparatus. The exploration time was set as 10 min (600 s) or the time required for the animal to eat all the food present in the apparatus.

To assess learning during exploration, total duration of each session was quantified over 7 days of exposure to the maze. The reference memory measure was evaluated by the number of entries into the arms without food. Each of these entries was counted as a reference memory error (RME). The measure of the working memory was the number of entries into the arms in which the rats had already consumed the food. Each of these re-entries was counted as a working memory error (WME). These two measures (RME and WME) were registered only in the last 2 days of exposure to the maze.

### Perfusion and Preparation of Biological Materials

After survival times of 15 and 30 days, animals were deeply (i.p.) anesthetized with xylazine hydrochloride (10 mg/kg) and ketamine hydrochloride (80 mg/kg). After reflex abolishment, animals were transcardially perfused with heparinized 0.9% phosphate-buffered saline (PBS) followed by a 4% paraformaldehyde solution in 0.1 M PB.

Brains were removed, immediately immersed in a 4% paraformaldehyde solution for 72 h and cryoprotected for 6 days in different gradients of sucrose-glycerol solutions in a 0.1 M phosphate buffer. Brains were then frozen in Tissue Tek, and the coronal sections were cut at a 30-μm thickness using a cryostat (Leica CM1850).

### Nissl Staining

To evaluate gross histology, representative sections from control, G15, and G30 animal groups were mounted onto gelatinized slides and submitted to Nissl staining. In short, slides were immersed in a decreasing concentration of alcohol solutions (100, 70, 50, and 20%) in distilled water for 1 min each, followed by immersion in a 0.1% thionin solution for 8 s. Slides were then immersed in distilled water and an increasing concentration of alcohol solutions (20, 50, 70, 90, and 100%) with an incubation time of 45 s each. They were then immersed in xylol for 5 min, mounted in Permount R (Fisher Scientific, Waltham, MA), and coverslipped for further analysis.

### Immunolabeling

To immunolabel cell bodies of differentiated neurons, we used an antibody against NeuN, a protein present in the nucleus of mature neurons (Mullen et al., 1992). To immunolabel immature neurons (neuroblasts), we used an antibody against doublecortin, a microtube-associated protein used as a specific marker for neuroblasts (Gleeson et al., 1999).

### Anti-NeuN Immunolabeling Protocol

Details of the immunolabeling protocol were reported in the previous publications (Thored et al., 2009). In short, sections were transferred to 24-well culture plates, rinsed three times in 0.1 M PBS (5 min each), and incubated with a 3% hydrogen peroxide solution for 15 min. Another wash battery in 0.1 M PBS was performed. Sections were incubated in citric acid solution in a water bath at 90°C for 20 min and were further allowed to cool for about 10 min at room temperature (20°C). Another wash battery with 0.1 M PBS was performed. Tissue permeabilization was performed with saponin solution for 10 min, followed by another washing battery with 0.1M PBS. The tissue was then incubated with a 10% BSA solution for 30 min and was subsequently incubated with primary antibodies overnight (anti-NeuN, LSBio^®^, Seattle, WA, United States, LS-C312122, 1:50).

On the second day, sections were labeled by an LSAB2 system-HRP (Dako^®^, Agilent, Santa Clara, CA, United States, K0675). They were rinsed three times with 0.1M PBS to remove the excess of primary antibody (5 min each) and were subsequently incubated with biotin for 30 min at 37°C, followed by another wash battery with PBS 0.1M before the incubation with streptavidin for 30 min at 37°C. Another washing battery with 0.1M PBS was made to remove streptavidin excess, and then, the revelation was made using the liquid DAB + substrate chromogen system (Dako^®^, K3468). Reaction time ranged from 04 to 05 min, and the reaction was stopped by washing in a 0.1M phosphate-buffered solution.

### Anti-DCX Immunolabeling Protocol

On the first day, sections were transferred to 24-well culture plates as for the previous antibody. They were rinsed two times in 0.1 M PBS (5 min each rinse), pretreated in 0.2 M boric acid (pH 9.0) at 60°C for 25 min, and allowed to cool for 20 min at room temperature (20°C). Sections were then incubated with 3% hydrogen peroxide in methanol solution for 20 min, rinsed three times with 0.05% PBS/TWEEN (5 min each), and incubated with 10% normal horse serum in PBS for 1 h. They were then incubated with a primary antibody overnight (anti-DCX, Santa Cruz Biotechnology, Dallas, TX, United States, Cat# sc-8066, RRID:AB_2088494, 1:100). On the second day, sections were rinsed three times in PBS/TWEEN and incubated with a secondary antibody biotinylated horse–antigoat (Vector Laboratories^®^, Burlingame, CA, United States, 1:100) for 2 h at room temperature. Another PBS/TWEEN washing battery was performed, and the sections were then incubated in an avidin-biotin-peroxidase complex (ABC kit, Vector Laboratories^®^) for 2 h. The sections were then rinsed four times in PBS/TWEEN (5 min each), and DAB was reacted with the reaction time ranging from 4 to 5 min. The reaction was stopped by washing the sections in a 0.1M phosphate-buffered solution.

All sections were mounted onto gelatinized slides, dehydrated in alcohol gradients, cleared in xylol, mounted onto slides using Permount, and coverslipped for further analysis.

### Qualitative Analysis

Sections of all the experimental groups were surveyed using a Zeiss Imager Z1 optical microscope. Representative images were obtained using a digital camera (Zeiss AxioCam HRc) attached to the microscope.

### Quantitative Analysis

Quantitative methods were based on our previous reports for hippocampal countings (Silva et al., 2013). DCX- and NeuN-positive cells from all experimental groups were counted using a graticule attached to the eyepiece of an optical microscope (Nikon, 50i). A X40 objective was used. The graticule counting area was 0.0625 mm^2^. At least four sections were counted per animal.

Countings were performed along with the full extent of the dentate gyrus and the initial area of the hippocampal hilus. In the dentate gyrus, the graticule was positioned in the granular cell layer. Due to the compact morphology of this hippocampal layer, the counting area was set as 0.01875 mm^2^, a fraction of the whole graticule area.

### Statistical Analysis

The Kruskal–Wallis test with Dunn’s posttest or one-way ANOVA with Tukey’s *post hoc* test was used for comparisons between groups. The significance level was set at *p* < 0.01, and the analysis was performed using the GraphPad Prism 7.0 software.

## Results

### Complete Unilateral Hippocampal Removal

**FIGURE 1:**
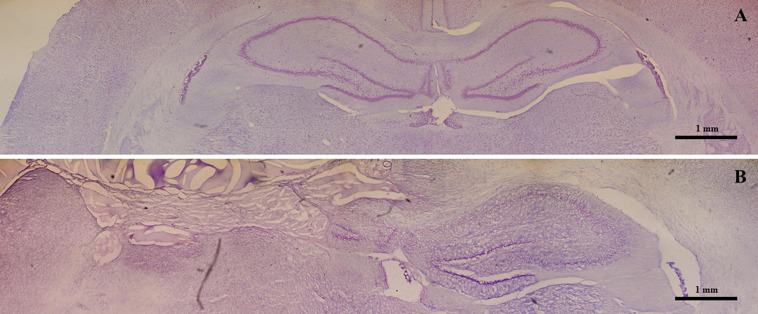
Effective experimental unilateral removal of rat hippocampus. Photo-reconstruction of a Wistar rat brain stained with 0.1% thionin. **(A)** Coronal section obtained from a control animal presenting with the right and left intact hippocampus. **(B)** A section obtained from an animal submitted to a left hippocampectomy. Note the absence of the left hippocampus.

Nissl staining revealed a normal hippocampal morphology in both control (Figure 1A) and hippocampectomized animals (Figure 1B). Hippocampectomy was successfully achieved as the left hippocampus was completely removed after surgery while the right hippocampus was preserved (Figure 1B).

### Unilateral Hippocampectomy Induces Increased Numbers of Migratory Neuroblasts in the Contralateral Hippocampus

**FIGURE 2:**
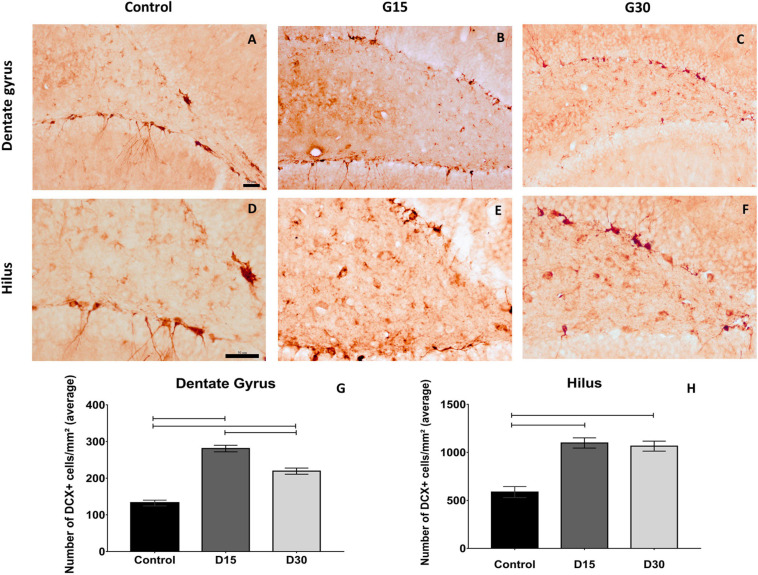
Compensatory increase in rat hippocampal neurogenesis after unilateral hippocampectomy. Immunolabeling for doublecortin (DCX) in coronal sections from animals belonging to all experimental groups (control, G15, and G30). There was an increase in the number of neuroblasts in both dentate gyrus **(B,C)** and hilus **(D–F)** compared to that of control **(A,D)**. This has been confirmed by the quantitative analysis [**(G,H)** Kruskal–Wallis with Dunn’s *post hoc* test (dentate gyrus); one-way ANOVA with Tukey’s posttest test (hilus), *p* < 0.01]. Scale bars: 50 μm; **(A–C)** objective X20; **(D–F)** objective X40.

The immunolabeling of migratory neuroblasts was performed using an anti-DCX antibody, which revealed DCX-positive cells (DCX+) in the dentate gyrus and hilus of animals belonging to all experimental groups. Nevertheless, quantitative differences in the number of DCX+ cells were observed between groups (Figure 2). There was an increase in the number of DCX+ cells in groups G15 (Figures 2B,E) and G30 (Figures 2C,F) compared to control (Figures 2A,D). These results were quantitatively confirmed (Figures 2G,H).

Statistical comparisons between groups revealed that the number of DCX+ cells were higher in G15 and G30 compared to those in control (Kruskal–Wallis/with Dunn’s *post hoc* test, *p* < 0.0001; Figures 2G,H). Comparisons between surgical groups revealed that the number of DCX+ cells in the dentate gyrus were higher in G15 compared to those in G30 (Figure 2G, *p* < 0.0001). This was not the case in the hilus (*p* > 0.0001; Figure 2H). In this region, the number of DCX+ cells remained equally elevated in both survival times. These results indicate an increased number of neuroblasts in the remaining hippocampus after experimental unilateral hippocampectomy.

### Increase in the Number of NeuN + Cells at 30 Days Post Hippocampectomy

**FIGURE 3:**
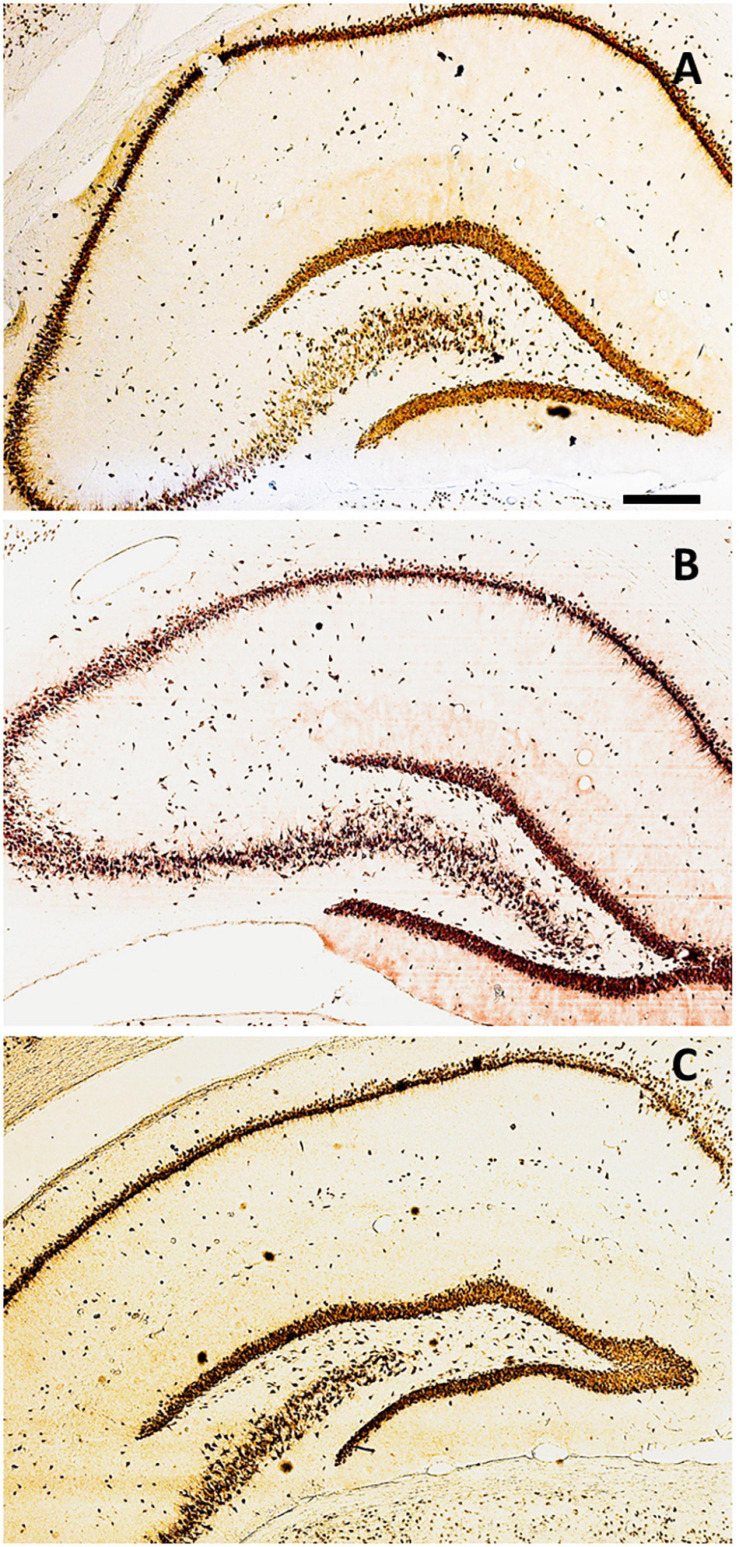
Distribution of NeuN+ cells along with the hippocampal areas. Coronal sections immunolabeled for NeuN in the control group (Gc) **(A)**, G15 **(B)**, and G30 **(C)**. Scale bar = 300 μm.

The immunolabeling of mature neurons was performed in animals after hippocampectomy using an anti-NeuN antibody, which revealed conspicuous hippocampal cytoarchitecture and morphology in all experimental groups (Figure 3). Immunolabeling was performed to infer some alteration on the normal hippocampal cytoarchitecture and morphology in the contralateral hippocampus after surgery.

**FIGURE 4:**
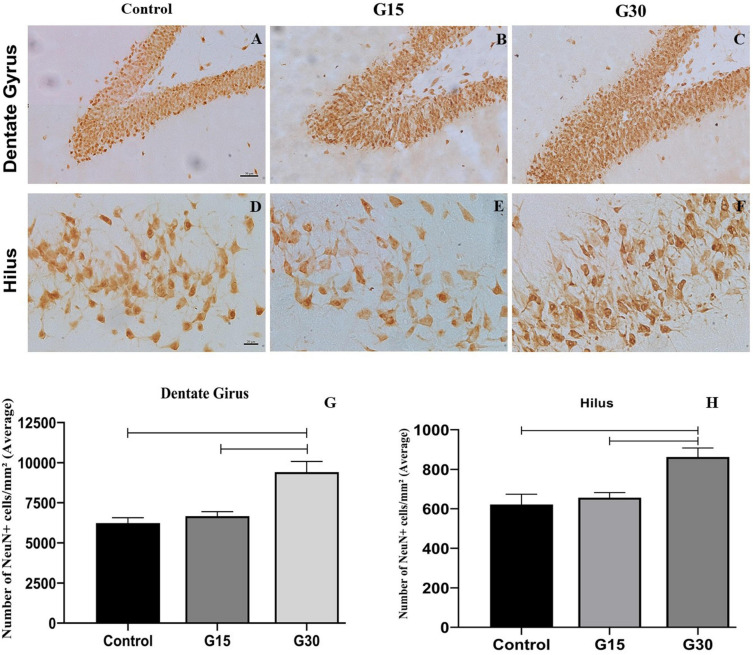
Compensatory increase in the number of NeuN+ cells in the hippocampal dentate gyrus following unilateral hippocampectomy. Immunolabeling for NeuN in coronal sections from animals belonging to all experimental groups (control, G15, and G30). There was an increase in the number of NeuN+ cells in both dentate gyrus **(A–C)** and hilus **(D–F)** at G30 compared to control **(A,D)**. This has been confirmed by the quantitative analysis [**(G,H)** Kruskal–Wallis with Dunn’s *post hoc* test (dentate gyrus); one-way ANOVA with Tukey’s posttest test (hilus), *p* < 0.01]. Scale bars: **(A–F)** 50 mm.

We have noted a thicker granule cell layer with a higher number of NeuN+ cells in both dentate gyrus (Figures 4A–C) and hippocampal hilus (Figures 4D–F), which parallels the increased number of DCX+ cells previously reported for this hippocampal region. These results were confirmed by quantitative analysis (Figures 4G,H).

To assess whether the increased number of DCX+ cells resulted in the incorporation of new adult neurons in the dentate gyrus in the remaining hippocampus, we performed countings of the number of NeuN+ cells in all experimental groups and survival times (Figures 4G,H). These countings revealed a statistically significant increase in the number of NeuN+ cells in both dentate gyrus (Figure 4G) and hilus (Figure 4H) at G30 post hippocampectomy compared to those at G15 (ANOVA-Tukey, *p* < 0.0005) and control (ANOVA-Tukey, *p* < 0.0001).

### Hippocampectomy Did Not Affect Learning in the Eight-Arm Radial Maze

All animals learned to collect food present at the end of the four arms of the apparatus, and no rat presented problems of adaptation to the maze. The maximum time that the animals remained in the apparatus was 10 min (600 s), the maximum allowed in the procedure, and the minimum recorded was 143 s. There was no preference for the ipsilateral or contralateral side in the eight-arm radial maze.

**FIGURE 5:**
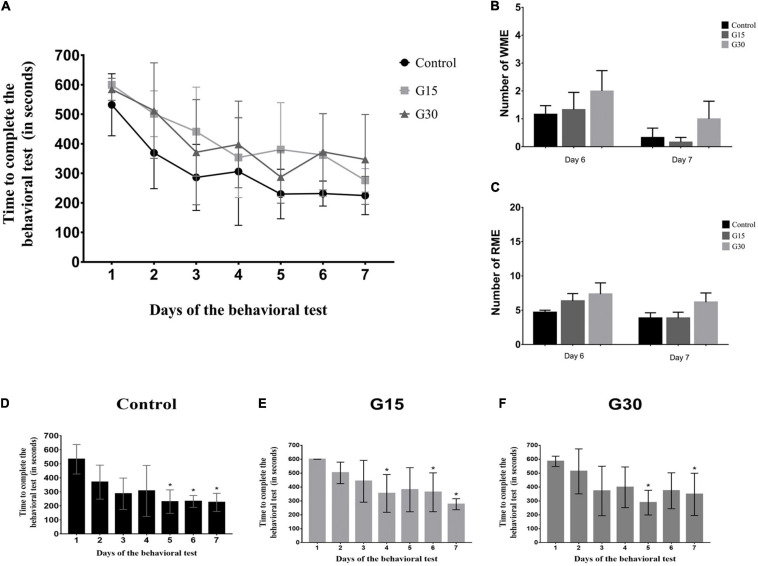
Quantitative data for learning and memory assessment. **(A)** The average time of exploration of the eight-arm radial maze per group in each experimental day. **(B)** Quantification of working memory errors (WMEs). **(C)** Quantification of reference memory errors (RMEs). **(D–F)** Times for test performance in each experimental day for all groups. No statistical differences were observed after comparisons (*p* > 0.05, Kruskal–Wallis with Dunn’s post hoc test). Asterisks in **(D–F)** indicate statistically significant improvement compared to day 1 (*p* < 0.05).

Table 1 shows the percentage of animals that consume all the food present in the apparatus before 10 min during the 7 days of an experiment for all experimental groups. All animals in the control group completed the task from the second day of exposure to the final day, except for day 4, when one animal did not complete (83%). The percentages were lower for the animals in the experimental groups. For G30, the percentage of animals that finished before time was gradually increased until 100% of the animals finished the test before the time of day 4 onward. For group G15, except for the first day, 83–100% of the animals finished the test before 10 min. These days can be related to surgery, but they were not statistically significant according to further analysis shown in Figure 5A.

**TABLE 1:** Percentage of animals that completed the eight-arm radial maze task consuming all the food present in the apparatus before 10 min in each of the 7-day experiment.

Group	Test days						
	Day 1	Day 2	Day 3	Day 4	Day 5	Day 6	Day 7
Control	33%	100%	100%	83%	100%	100%	100%
G15	0%	83%	100%	83%	83%	100%	100%
G30	33%	50%	67%	100%	100%	100%	100%

*The total number of animals exposed per day in each group was 6 (*n* = 6 per group).*

### Time of Exploration in the Eight-Arm Radial Maze Decreases for All Groups During Behavioral Procedures

The average time that each group remained in the maze was quantified in all 7 days, and a decrease of this variable for all groups (Figure 5A) was observed as a function of time, indicating a faster execution of the task, that is, in the consummation of the four pieces of food. Although the shortest time was observed for the subjects in the Gc on all days, comparisons between groups did not present a significant statistical difference (Figure 5A; ANOVA-Tukey, *p* > 0.05).

To evaluate whether the decrease in intragroup time was significant, we compared the exposure time to the maze on the first day with time on the other days. In the Gc, there was a significant difference between the time on day 1 and the time on days 5–7 (Figure 5D); in the G15 group, there was a difference between the time on day 1 and days 4, 6, and 7 (Figure 5E), and in G30 group, there was a difference between day 1 and days 5 and 7 (Figure 5F). Therefore, in general, a significant difference was observed in the reduction of time to perform the task between days 1 and 7 in all groups, including control and hippocampectomized animals.

### Hippocampectomy Does Affect Animal Performance on Working and Reference Memory Behavioral Tests

As to the memory measures, the analysis of the WMEs and the RMEs of the animals in the maze was performed through the comparison between the control and the surgical groups at the sixth and seventh days of the experiment. The comparison of the EMT and RME between the groups did not show significant differences on both days analyzed (Figures 5B,C). These results reinforce the idea that learning in the eight-arm radial maze was not permanently affected by surgery.

## Discussion

Unilateral hippocampectomy surgery performed on human patients with refractory TLE promotes the unilateral removal of the hippocampus affected by the pathology and the adjacent cortical area (Jobst and Cascino, 2015; Boling, 2018). This surgery is an alternative treatment for those patients who do not get control of seizures with drug treatment and is reported as effective in the control of recurrent epileptic seizures (Liao et al., 2018).

In this study, we developed an experimental model of unilateral hippocampectomy in adult rats to simulate the human surgery used to treat refractory TLE. The results show that there was complete removal of the left hippocampus with the preservation of the right one. The implementation of this experimental model of unilateral hippocampectomy allowed us to study the possible neuroplastic events in the contralateral intact hippocampus.

Following surgery, there was an increase in the number of neuroblasts in both dentate gyrus and hilus of the contralateral hippocampus at 15 and 30 days postsurgery, with the incorporation of adult granular neurons derived from adult-born neuroblasts at G30. In addition, behavioral tests did not reveal any memory and learning deficits, which parallels the human findings.

### Increased Adult Neurogenesis May Occur in the Contralateral Hippocampus as a Compensatory Mechanism to Avoid Later Cognitive Impairment

Experimental hippocampectomy induced the increased neurogenesis in the contralateral hippocampus up to 1 month after surgery. This is likely a compensatory neuroplastic event to avoid the loss of hippocampal function. This is supported by the fact that animals do not present any important cognitive impairment despite complete unilateral hippocampectomy. The same results are present in humans after unilateral hippocampal removal to treat refractory TLE (Jobst and Cascino, 2015; Boling, 2018).

The mechanisms that trigger compensatory events in the remaining hippocampus are unknown. It has been established that adult neurogenesis is dramatically changed by pathological alterations, including stroke, trauma, epilepsy, and other neural disorders (Jin et al., 2006; Lazarov and Marr, 2010; Zhong et al., 2016). Epilepsy and ischemic stroke induce an increase in hippocampal neurogenesis (Zhong et al., 2016; Koh and Park, 2017), while the reduction of hippocampal neurogenesis occurs during Alzheimer’s disease, even in humans (Moreno-Jiménez et al., 2019).

We have reported that the number of neuroblasts was higher in G15 compared to those in G30 in the rat dentate gyrus. These differences may be the result of a neuroblast maturation at 30 days, which normally induces the reduction of DCX expression. The data suggest that this was exactly the case, as there was an increase in the number of NeuN+ cells in both dentate gyrus and hilus at G30. There was no difference in the number of neuroblasts between G15 and G30 in the hippocampal hilus, which suggests a differential pattern of neurogenic events in this region.

In the hilar region, it seems that neuroblast migration continues for a longer time or the level of maturation of adult-born cells is different from the dentate gyrus. The presence of neuroblasts in the hippocampal hilus may represent the ectopic migration of adult-born cells to this hippocampal region. This has been reported after status epilepticus (Yang et al., 2009) and stroke (Woitke et al., 2017).

There is a continuous formation of neuroblasts in the adult hippocampus even in older individuals (Boldrini et al., 2018; Moreno-Jiménez et al., 2019). This precludes an important function for adult neurogenesis on the normal hippocampal function. It has been established that adult-born neurons possess a pivotal role in pattern separation (Sahay et al., 2011), reversal learning, and cognitive flexibility, functions related to the hippocampal activity (Anacker and Hen, 2017). Loss of these functions may underlie some mood disorders, including anxiety and depression (Anacker and Hen, 2017). The compensatory events in this study described may play an important role in the avoidance of these pathological events.

### Incorporation of Adult-Born Cells Into the Granule Layer May Occur in the Contralateral Hippocampus

We searched for alterations on the normal hippocampal cytoarchitecture after unilateral hippocampal removal. In general, we found no major alterations of density and cellular dispersion between groups.

In the dentate gyrus, we have found an increased number of NeuN+ cells in the granule cell layer in G30, which suggests that adult-born cells may be integrated into the granule cell layer in the contralateral hippocampus. This is likely a fundamental compensatory event to avoid the loss of hippocampal function following unilateral hippocampectomy. Such an event might occur in human patients submitted to hippocampal removal to treat refractory TLE. This hypothesis is supported by a recent finding of German researchers that, using MRI, reported a significant increase in the contralateral right hippocampal volume following the resection of the left anterior medial temporal lobe at 3 months after surgery (Pajkert et al., 2020). Future studies, using functional MRI adapted to visualize human neurogenesis, might confirm the present experimental results.

### Experimental Unilateral Hippocampectomy Does Not Induce Cognitive Impairment, Which Parallels Human Patients Who Have Undergone Surgery

The eight arms allowed the evaluation of inter and intragroup learning by means of measures of the average time spent in the maze and the percentage of animals that completed the task in the course of the days. Animals submitted to surgery completed the behavioral tests after a delay in early time points. This may be a consequence of the surgery, but the results were not statistically significant. There was no side preference in this study, as the animals traveled both sides of the radial maze. Despite the good correlation between the neuropathological and behavioral data, we cannot completely rule out that a small sample size effect (*N* = 6 per survival time) influenced the absence of the statistical significance for the variable time to complete the test. In future studies, statistical power can be increased by using a higher sample size.

A group of non-operated animals was used as a control because we wanted to evaluate how the Wistar rats naturally demonstrated their learning patterns, without the interference of subsequent surgery stressors that could alter the intrinsic behavior and learning patterns of their species. However, a group that simulated surgery is also an important standard of analysis, and it is in the future planning to carry out tests in a group with this experimental design.

The number of days of exposure to eight arms varies in the literature; Chan et al. (2004) used 12 days of maze exposure, while Kemse et al. (2018) and Rathod et al. (2014, 2015) have shown a change in the behavioral patterns with 5 days of exposure to the maze. In the present study, 7 days of exposure appears to have been an adequate choice, as the time was suitable for a minimum survival time (15 days) and the allowed physical recovery of the animals after the surgical procedure.

Learning and memory are fundamental cognitive functions of organisms, and impairments to these functions can significantly affect the quality of life. The reference memory is a type of memory that has a longer duration, and its consolidation is carried out by the hippocampal formation, which can be affected by any damage to this structure; working memory allows the temporary storage of information being used during the execution of certain tasks and being generally eliminated at the end of the task execution (Chan et al., 2004; Squire and Wixted, 2011).

Anterograde amnesia (inability to consolidate new memories) is described in individuals who have bilateral damage in the temporal lobe area (Squire, 2009; Squire and Wixted, 2011). However, unilateral hippocampectomy surgery is reported to be safe and generally not associated with cognitive impairment related to processing and memory recall in patients (Wiebe et al., 2001; Vojtěch et al., 2012; Jobst and Cascino, 2015; Mathon et al., 2015; Anyanwu and Motamedi, 2018). This is in agreement with the experimental results described in this study, as rats submitted to hippocampectomy did not present any learning and memory deficits.

Considering that the hippocampus is an important center of integration and consolidation of memories, the absence of cognitive impairment is likely explained by the fact that the contralateral hippocampus continues to perform its activity, compensating for the absence of the removed hippocampus. In this study, we propose that compensatory neurogenic events are fundamental neuroplastic mechanisms that contribute to the absence of learning and memory impairment.

It can be argued that additional behavioral tests, including the Nobel object recognition (NOR) test can be used to strengthen the behavioral analysis. NOR test is also used to assess the acquisition of new memory repertoires in the rat. Despite it being simpler, the results obtained also indicated repertoire acquisition *via* learning and memory, which is suitable for the analysis. In the follow-up studies, we can apply different behavioral tests in different and longer survival times.

## Conclusion

We conclude that unilateral hippocampectomy induces a compensatory neurogenic effect in the contralateral hippocampus. This may underlie the absence of a significant cognitive impairment after unilateral hippocampal removal. The results parallel the findings in human patients submitted to unilateral hippocampectomy to treat refractory TLE. These results also establish an experimental model for behavioral evaluation and the analysis of possible compensatory neural processes induced by surgery.

## Data Availability Statement

The raw data supporting the conclusions of this article will be made available by the authors without undue reservation.

## Ethics Statement

The animal study was reviewed and approved by the Ethics Committee on Research with Experimental Animals of the Federal University of Pará (CEUA-UFPA) (approval number 6552280616).

## Author Contributions

WG-L and SL developed the paper rationale and wrote the manuscript. GC and FG performed surgical and immunohistochemical experiments. EF, AB, and AP performed behavioral analysis. All authors contributed to the article and approved the submitted version.

## Conflict of Interest

The authors declare that the research was conducted in the absence of any commercial or financial relationships that could be construed as a potential conflict of interest.

## Publisher’s Note

All claims expressed in this article are solely those of the authors and do not necessarily represent those of their affiliated organizations, or those of the publisher, the editors and the reviewers. Any product that may be evaluated in this article, or claim that may be made by its manufacturer, is not guaranteed or endorsed by the publisher.
